# Pharmacologic strategies to prevent hemodynamic changes after intubation in parturient women with hypertensive disorders of pregnancy: A systematic review and network meta-analysis protocol

**DOI:** 10.1097/MD.0000000000018454

**Published:** 2019-12-20

**Authors:** Sang Won Yoon, Hyun Kang, Geun Joo Choi

**Affiliations:** Department of Anesthesiology and Pain Medicine, Chung-Ang University College of Medicine, Seoul, Republic of Korea.

**Keywords:** hemodynamics, hypertension, network meta-analysis, pregnancy-induced, systematic review

## Abstract

**Background:**

Hypertensive disorders of pregnancy are major health issues affecting mothers and infants. General anesthesia is inevitable for certain mothers with these conditions and they may exhibit pronounced hemodynamic instability, especially during induction and airway manipulation. The aims of this study are to combine direct and indirect comparisons of the efficacies of different medications used in attenuating reflex hemodynamic responses and generate intervention ranking by network meta-analysis (NMA).

**Methods:**

A systematic and comprehensive search will be performed using MEDLINE, EMBASE, the Cochrane Central Register of Controlled Trials (CENTRAL), and Google Scholar databases, beginning from their inceptions to November 2019. Only randomized clinical trials evaluating the efficacy and safety of pharmacologic interventions for preventing hemodynamic changes after intubation in parturient women with hypertensive disorders of pregnancy will be included. The primary endpoints will be maximal mean arterial pressure (MAP) and maximal heart rate after intubation. Maximal systolic arterial pressure (SAP), diastolic arterial pressure (DAP), and maternal/fetal blood gas analysis, as well as safety issues, including Apgar score at 1 minute and 5 minutes after delivery, will be also assessed.

We will conduct both pairwise meta-analysis and network meta-analysis. We will use surface under the cumulative ranking curve (SUCRA) values and rankograms to present the hierarchy of pharmacologic interventions. A comparison-adjusted funnel plot will be used to assess the presence of small-study effects. The quality of the studies included will be assessed using the risk of bias tool 2.0. All statistical analyses will be performed using Stata SE, version 15.0 (StataCorp, College Station, TX).

**Results:**

The results of this systematic review and NMA will be published in a peer-reviewed journal.

**Conclusion:**

This NMA will enable us to determine the order of effectiveness and safety of pharmacological interventions used in attenuating hemodynamic responses for mothers with hypertensive disorders of pregnancy.

**Trial registration number:**

CRD42019136067.

## Abstract 2

Supplemental Digital Content is available in the text

## 1 Introduction

Hypertensive disorders of pregnancy, including preeclampsia and gestational hypertension, account for 10% of complications in pregnancy worldwide and are major health issues affecting mothers and infants. Preeclampsia is characterized by the onset of high blood pressure with proteinuria or significant end-organ dysfunction, and women with preeclampsia are at a greater risk for lethal medical complications such as HELLP syndrome.^[1]^ Cesarean section may often be required for these hypertensive women and anesthesiologists should pay meticulous attention when managing them. Generally, regional anesthesia is favored over general anesthesia since it circumvents airway management, use of neuromuscular blocking agents, and increased hemodynamic instability.^[2]^ However, general anesthesia is inevitable for certain mothers due to coagulopathy and thrombocytopenia and because they may exhibit pronounced hemodynamic instability especially during induction and airway manipulation. Laryngoscopy and tracheal intubation stimulate sympathetic nervous system and result in reflex hemodynamic response such as hypertension, tachycardia, and arrhythmia.^[3]^

Increased morbidity and mortality in both mothers and infants could result from hypertension after airway manipulation, which is related to elevated intracranial pressure, cerebral hemorrhage, cardiac failure and pulmonary edema.^[4]^ Therefore, opioids or anti-hypertensive drugs have been adopted by anesthesiologists to attenuate reflex hemodynamic responses after intubation.^[5]^ Drugs such as fentanyl, alfentanil, remifentanil, magnesium sulfate, and nitroglycerine have been used and their effectiveness has been compared. In addition, different dosages of the same drug were compared to investigate optimal dosage without intraoperative hypotension.^[6]^

Although there are several studies comparing drugs used to alleviate hypertension and tachycardia after intubation, few target hypertensive disorders of pregnancy. Moreover, the effects of drugs used to alleviate hemodynamic instability on fetal pH or Apgar scores remain ambiguous. We plan to review all the randomized controlled trials comparing pharmacological interventions to attenuate hemodynamic instabilities in mothers with hypertensive disorders of pregnancy, including preeclampsia. The aims of this study are to combine direct and indirect comparisons of the efficacy and safety of different medications used in attenuating reflex hemodynamic responses, and generate intervention ranking by network meta-analysis (NMA).

## 2 Methods

### 2.1 Protocol design and registration

This protocol was developed following the Preferred Reporting Items for Systematic Review and Meta-analyses (PRISMA) extension statement for NMA.^[7]^ The protocol for this systematic review and NMA has been registered with the International Registration of Prospective Systematic Reviews (PROSPERO network; registration number CRD42019136067).

### 2.2 Eligibility criteria

#### 2.2.1 Type of studies

Peer-reviewed randomized controlled trials will be eligible for inclusion. No language or date restriction will be applied. Review articles, case reports, case series, letters to the editor, commentaries, proceedings, laboratory science studies, and any other non-relevant studies will be excluded from this analysis.

#### 2.2.2 Participants

All parturient women with hypertensive disorders of pregnancy undergoing cesarean section under general anesthesia will be included. Those undergoing surgery under regional anesthesia will be excluded.

#### 2.2.3 Interventions and comparisons

Pharmacological interventions administered for preventing hemodynamic change after intubation for general anesthesia will be included. We will include trials comparing one or more pharmacological intervention(s) for the prevention of hemodynamic changes against no treatment or placebo.

#### 2.2.4 Outcomes

##### 2.2.4.1 Effectiveness

The primary endpoints will be maximal mean arterial pressure (MAP), and heart rate after intubation. Maximal systolic arterial pressure (SAP), diastolic arterial pressure (DAP) and maternal/fetal blood gas parameters will also be assessed.

##### 2.2.4.2 Safety

Apgar score at 1 minute and 5 minutes after delivery will be assessed.

### 2.3 Information sources

#### 2.3.1 Electronic search

A search will be performed in the MEDLINE, EMBASE, Cochrane Central Register of Controlled Trials (CENTRAL), and Google Scholar databases, beginning from their inception to November 2019, using search terms such as and related to “pregnancy induced hypertension”, “cesarean section” and “hemodynamic change”. The search strategy, which includes a combination of free text, Medical Subject Heading (MeSH), and EMTREE terms, is outlined in the Supplemental Digital Content.

Additional relevant articles will be identified by scanning the reference lists of the articles found during the original search and meta-analyses. Reference lists will be imported into Endnote software (Thompson Reuter, CA), and duplicate articles will be removed.

#### 2.3.2 Study selection

The titles and abstracts identified through the search strategy described above will be scanned independently by 2 of the study's authors (SWY and GJC). To minimize data duplication as a result of multiple reporting, papers from the same author will be compared. For studies determined to be eligible based on the title or abstract, the full paper will be retrieved. All abstracts that cannot provide sufficient information regarding the eligibility criteria will be selected for full-text evaluation. Any potentially relevant studies chosen by at least one of the authors will be retrieved and their full-text versions will be evaluated. In the second phase, the same reviewers will independently evaluate the full-text articles and make their selection in accordance with the eligibility criteria.

The articles that will meet the inclusion criteria will be assessed separately by two of the study's authors (SWY and GJC), and any discrepancies will be resolved through discussion. In cases where an agreement cannot be reached, the dispute will be resolved with the help of a third investigator (HK). A flow diagram for the search and selection process that follows the preferred reporting items for systematic reviews and meta-analysis (PRISMA) guidelines will be developed.

#### 2.3.3 Data extraction

All interrelated data from the included studies will be independently extracted and entered into a standardized form by 2 of the study's authors (SWY and GJC) and will then be cross-checked. Any discrepancy will be resolved through discussion. If an agreement cannot be reached, the dispute will be resolved with the aid of a third investigator (HK).

The standardized extraction form includes the following items, and the data will be extracted independently by 2 of the study's authors (SWY and GJC):

1) title;
2) authors;
3) name of journal;
4) publication year;
5) study design;
6) registration of clinical trial registry;
7) competing interests;
8) country;
9) risk of bias;
10) number of patients in study;
11) kinds and doses of drugs compared;
12) age of parturient women;
13) weight of parturient women;
14) height of parturient women;
15) duration of anesthesia;
16) American Society of Anesthesiologists’ physical status score;
17) inclusion criteria;
18) exclusion criteria;
19) drug used for induction;
20) kinds of muscle relaxant;
21) kinds of reversal agents;
22) maximal SAP;
23) maximal MAP;
24) maximal DAP;
25) maximal HR and 26) maternal and fetal ABGA.

If information is missing, an attempt will be made to contact the study authors to obtain the relevant information. If data are presented as figures rather than numbers, the open source software Plot Digitizer (version 2.6.8; http://plotdigitizer.sourceforge.net) will be used to extract the numbers. For studies reporting the results from different doses, the groups will be combined in order to avoid a unit of analysis error.

The degree of agreement between the 2 independent data extractors will be computed using kappa statistics to measure the difference between the observed and expected agreements; namely, whether they were random or by chance. Kappa values will be interpreted as:

1) less than 0: less than chance agreement;
a. to 0.20: slight agreement;
2) 0.21 to 0.40: fair agreement;
3) 0.41 to 0.60: moderate agreement;
4) 0.61 to 0.80: substantial agreement; and
5) 0.8 to 0.99: almost perfect agreement.^[8]^

### 2.4 Study quality assessment

The quality of the studies will be independently assessed by 2 of the study's authors (GJC and HK), using the Revised Cochrane risk of bias tool for randomized trials (RoB 2.0).^[9]^ The risk of bias (ROB) will be evaluated by considering the following five potential sources of bias:

1) bias arising from the randomization process;
2) bias due to deviations from the intended interventions;
3) bias due to missing outcome data;
4) bias in measurement of the outcome; and
5) bias in selection of the reported result.

Then, we will evaluate the overall risk of bias judgment according to these domain-level judgments. The methodology for each domain will be graded as “Low risk of bias,” “Some concerns,” and “High risk of bias,” which reflects a low risk of bias, some concerns, and a high risk of bias, respectively.^[9]^

### 2.5 Statistical analysis

Ad-hoc tables will be designed to summarize the data from the included studies, show their key characteristics, and answer any important questions related to the aim of this review. If a trial result is presented with zero events in one group, the event rate will be artificially inflated by adding 0.5. After the data have been extracted, reviewers will determine whether a meta-analysis is possible. For this, we will evaluate the heterogeneity and transitivity assumptions by examining the comparability of the patients’ eligibility criteria, pertinent patients’ demographics, study design, and the risk of bias (all degrees of bias versus removing a “High risk of bias” evaluated in overall risk of bias judgment) as potential treatment-effect modifiers across comparisons.^[10]^ We will note the methodological differences between the studies that could influence the outcome measurement, as well as any concerns related to the transitivity assumption or methodological heterogeneity.

When the treatment nodes form a connected network of evidence, we will perform a network meta-analysis. A multiple treatment comparison network meta-analysis (NMA) is a generalization of meta-analysis methods that includes both the direct randomized controlled trial (RCT) comparisons as well as the indirect comparisons of treatments. An NMA based on a frequentist framework will be performed using the NMA graphical tools by Chaimani et al.^[11]^ Given the clinical and methodological heterogeneity of the populations and methods among the included trials in the NMAs, we will use the random-effects model in our primary analysis.

A network plot linking all the included pharmacologic agents will be formed to indicate the type of pharmacologic agents, number of patients under different pharmacologic agents, and number of pair-wise comparisons. In the network plot, nodes will show the pharmacologic agents being compared, and edges will show the available direct comparisons between the pharmacologic agents. Each drug, as well as each combination of drugs, will be treated as a node in this network. Nodes and edges will be weighted according to the number of patients and studies, respectively.

We will examine the consistency of the total network through both global and local tests of inconsistency. We will evaluate the global consistency assumption using the design-by-treatment interaction model.^[12]^ We will also evaluate each closed loop in the network in order to examine the local inconsistency between the direct and indirect effect estimates for the same comparison. In each loop, we will estimate the inconsistency factor (IF) as the absolute difference (with 95% confidence interval (CI) and a z-test) between the direct and indirect estimates for each paired comparison in the loop. The IF is the logarithm of the ratio of two odds ratios (RoR) from the direct and indirect evidence in the loop; RoR values close to 1 indicate that the two sources are in agreement.

We will also show the relative treatment effects between all active medications in ranked forest plots. The mean summary effects with CIs will be presented together with their predictive intervals (PrIs) to facilitate the interpretation of the results in light of the magnitude of heterogeneity. PrIs provide an interval that is expected to encompass the estimate of a future study. We will not adjust for multiple comparisons in successive NMAs, as we are not interested in establishing the superiority or inferiority of particular comparisons.

A rankogram and cumulative ranking curve will be drawn for each pharmacologic agent. A rankogram plots the probabilities for treatments to assume any of the possible ranks. It is the probability that a given treatment ranks first, second, third, and so on, among all the treatments evaluated in the NMA. We will use the surface under the cumulative ranking curve (SUCRA) values to present the hierarchy of the interventions. SUCRA is a relative ranking measure that accounts for the uncertainty in the treatment order, meaning it accounts for both the location and the variance of all relative treatment effects.^[13]^ A higher SUCRA value is regarded a better result for an individual intervention. When ranking treatments, the closer the SUCRA value is to 100%, the higher the treatment ranking is. We will test for small study effects and publication bias using the comparison-adjusted funnel plot.^[14]^

If clinical and methodological heterogeneity between the study arms are found to be substantial, or only two groups are compared in certain outcomes, a pairwise meta-analysis will be conducted to generate summary estimates and to assess the statistical heterogeneity across the included studies. The summary estimates will be reported as mean differences, standardized mean differences, or relative risks (RRs), as appropriate, with corresponding 95% confidence intervals (CIs). The heterogeneity between the studies will be assessed using the Cochran's Q and the Higgins I^2^ statistics. A level of 10% significance (*P* < .10) in the Chi^2^ statistic or an I^2^ greater than 50% will be regarded considerable heterogeneity, and the data will be analyzed using the Mantel–Haenszel random-effect model. Otherwise, the Mantel–Haenszel fixed-effect model will be applied.^[15]^

Publication bias will be assessed using the Begg funnel plot and the Egger test. If the funnel plot is asymmetrical *or* the *P* value is found to be <.1 by the Egger's test, the presence of a publication bias will be considered, and trim and fill analyses will be performed.

If included studies are fewer than 10, publication bias will not be assessed.

If the transitivity assumption cannot be adequately met, a descriptive summary of the study findings will be presented. If an inconsistency in the entire network or a local inconsistency is suspected, we will conduct sensitivity analyses to evaluate the reason for the inconsistency, as well as the influence of individual studies on the overall effect estimate by excluding one study at a time from the analysis. All statistical analyses will be performed using Stata SE, version 15.0 (StataCorp, College Station, TX).

### 2.6 Evidence synthesis

Based on the results of the NMA for the RCTs, the overall quality of evidence for each outcome assessed will be rated using the guidelines developed by the Grading of Recommendations Assessment, Development, and Evaluation working group. These guidelines are designed to rate the quality of the effect estimates derived from an NMA and uses a sequential assessment of the evidence quality, followed by an assessment of the risk-benefit balance and a subsequent judgment on the strength of recommendations.^[16]^ We will use a 4-step process:

1) present direct and indirect treatment estimates (mean differences, standardized mean differences, or RRs with 95% CIs);
2) rate the quality of the direct and indirect treatment estimates;
3) present the NMA estimates (pool of direct and indirect estimates, mean differences, standardized mean differences, or RRs with 95% CIs); and
4) rate the quality of the NMA estimates.

### 2.7 Ethics and dissemination

#### 2.7.1 Ethical issues

This systematic review does not require ethical approval or the patients’ informed consent because there will be no direct contact with individual patients. Only previously published data will be included in the review.

#### 2.7.2 Publication plan

This systematic review will be published in a peer-reviewed journal and will be disseminated electronically and in print.

## 3 Discussion

General anesthesia in women with hypertensive disorders of pregnancy is associated with increased maternal and perinatal mortality; therefore, anesthesiologists have adopted different medical interventions to avoid hemodynamic instability during laryngoscopy and tracheal intubation. The objective of this systematic review and NMA is to determine the ranking of effectiveness of pharmacological interventions adopted to attenuate hypertension and tachycardia. We also aim to analyze the effects of drugs on fetal outcome such as fetal pH and Apgar scores.

To our knowledge, there have been several attempts to compare and address effects of pharmacological interventions used to attenuate hemodynamic instability. However, the results were inconsistent, and effectiveness of drugs has remained controversial. Furthermore, there was no systematic review and meta-analysis that specifically focused on women with hypertensive disorders of pregnancy receiving Cesarean section under general anesthesia. Therefore, we believe that this NMA will enable us to determine the order of effectiveness and safety of pharmacological interventions for mothers and fetuses. In addition, the outcome of this NMA will necessitate supplementary studies for further analysis of optimal dosages of drugs that prevent hypotension after induction.

The expected limitations of this NMA are as follows. First, the time and method of drug administration varies in the included studies, and sufficient onset time of drugs could not have been reached before intubation. Second, different dosage and injection methods (bolus or continuous infusions) of the same drug could affect the result of this NMA and subgroup and sensitivity analysis may be necessary. Lastly, poor fetal outcome in some studies could be due to severe prematurity or pre-existing conditions of fetuses rather than the compared drug, and this should be considered when evaluating interventions.

## Author contributions

**Conceptualization:** Sang Won Yoon, Hyun Kang, Geun Joo Choi.

**Data curation:** Sang Won Yoon, Hyun Kang, Geun Joo Choi.

**Formal analysis:** Hyun Kang, Geun Joo Choi.

**Funding acquisition:** Hyun Kang.

**Investigation:** Sang Won Yoon, Hyun Kang, Geun Joo Choi.

**Methodology:** Sang Won Yoon, Hyun Kang, Geun Joo Choi.

**Project administration:** Geun Joo Choi.

**Resources:** Sang Won Yoon.

**Supervision:** Hyun Kang.

**Visualization:** Hyun Kang.

**Writing – original draft:** Sang Won Yoon, Geun Joo Choi.

**Writing – review & editing:** Hyun Kang.

Hyun Kang orcid: 0000-0003-2844-5880.

## Supplementary Material

Supplemental Digital Content
