# Supplementary material for: Pharmacologic strategies to prevent hemodynamic changes after intubation in parturient women with hypertensive disorders of pregnancy: A systematic review and network meta-analysis protocol
Source: Medicine (Baltimore). 2019 Dec 20;98(51):e18454. doi: 10.1097/MD.0000000000018454 (PMC6940058; doi:10.1097/MD.0000000000018454)
Supplement: Supplemental Digital Content [file medi-98-e18454-s001.docx]

**Appendix**

**Medline**

1. randomized controlled trial.pt
2. randomized controlled trial$.mp
3. controlled clinical trial.pt
4. controlled clinical trial$.mp
5. random allocation.mp
6. exp double-blind method/
7. double-blind.mp
8. exp single-blind method/
9. single-blind.mp
10. or/1-9
11. clinical trial.pt
12. clinical trial$.mp
13. exp clinical trial/
14. (clin$ adj25 trial$).mp
15. ((singl$ or doubl$ or tripl$ or trebl$) adj25 (blind$ or mask$)).mp
16. random$.mp
17. exp research design/
18. research design.mp
19. or/11-18
20. 10 or 19
21. Case report.tw.
22. Letter.pt.
23. Historical article.pt.
24. Review.pt.
25. or/21-24
26. 20 not 25
27. exp intubation, intratracheal/
28. (intub$ or ((airway or respiratory tract) adj3 manage$)).mp.
29. 27 or 28
30. Intratracheal.mp
31. Endotracheal.mp
32. Tracheal.mp
33. Or/30-32
34. Intubating.mp.
35. Intubation.mp.
36. tube.mp.
37. Or/34-36
38. 26 and 37
39. exp cesarean section/
40. caesarean.mp.
41. cesarean.mp.
42. c-sec.mp.
43. c-section.mp.
44. abdominal and deliver$
45. or/39-44
46. 38 and 45
47. exp hypertension, Pregnancy induced/
48. pregnancy induced hypertension.mp.
49. gestational hypertens$.mp
50. exp eclamsia/
51. exp pre-eclamsia/
52. exp HELLP syndrome/
53. exp hypertension/
54. or/47-53
55. 46 and 54

EMBASE

1. randomi?ed controlled trial$.mp.
2. 'controlled clinical trial (topic)'/exp
3. controlled AND clinical AND trials
4. controlled clinical trial$.mp.
5. 'randomization'/exp
6. 'random allocation'/exp
7. random allocation.mp.
8. double-blind.mp.
9. single-blind.mp.
10. #1 OR #2 OR #3 OR #4 OR #5 OR #6 OR #7 OR #8 OR #9
11. 'clinical trial (topic)'/exp
12. clinical AND trial$.mp.
13. random$.mp.
14. rct
15. #11 OR #12 OR #13 OR #14
16. #10 OR #15
17. 'case study'/exp
18. 'case report'/exp
19. 'abstract report'/exp
20. 'letter'/exp
21. #17 OR #18 OR #19 OR #20
22. #16 NOT #21
23. ‘respiratory tract intubation’/exp
24. Intubation
25. Intubating
26. ‘Endotracheal tube’/exp
27. #23 OR #24 OR #25 OR #26
28. #22 AND #27
29. ‘cesarean section’/exp
30. caesarean.mp.
31. cesarean.mp.
32. c-sec.mp.
33. c-section.mp.
34. abdominal and deliver$
35. #29 OR #30 OR #31 OR #32 OR #33 OR #34
36. #28 AND #35
37. ‘maternal hypertension’/exp
38. pregnancy induced hypertension.mp.
39. gestational hypertens$.mp.
40. eclamsia.mp
41. pre-eclamsia.mp.
42. HELLP.mp.
43. #37 OR #38 OR #39 OR #40 OR #41 OR #42
44. #36 AND #43
